# Spatiotemporal Diurnal Modulation Characteristic of Wind Speed and Power Generation Revealed by Its Measured Data Processing

**DOI:** 10.1155/2022/5722770

**Published:** 2022-03-31

**Authors:** Jie Wan, Kun Yao, Guorui Ren, Ke Han, Qi Wang, Jilai Yu

**Affiliations:** ^1^Laboratory for Space Environment and Physical Sciences, Harbin Institute of Technology, Harbin 150001, China; ^2^School of Electrical Engineering &Automation, Harbin Institute of Technology, Harbin 150001, China; ^3^The State Key Laboratory of Alternate Electrical Power System with Renewable Energy Sources (North China Electric Power University), Beijing 102206, China; ^4^School of Computer and Information Engineering, Harbin University of Commerce, Harbin 150028, China

## Abstract

Atmospheric turbulence is an intrinsic factor that causes uncertainty of wind speed and its power generation by wind turbine. The research of atmospheric turbulence characteristics of wind farms can be used to reduce this uncertainty. In this paper, enough measurement data getting from actual wind farms is used for information processing to quantitatively analyze the daily variation of wind speed and its power output characteristics. Furthermore, the concept of spatiotemporal diurnal modulation characteristics of atmospheric turbulence is proposed with a global scope, which is an intrinsic property of wind. Besides the daily variation characteristics, the average hourly wind speed has a short-term modulation effect on its turbulence and provides a modulation characteristic on wind speed uncertainty. Moreover, the long-term modulation process is affected by seasonal and regional factors, indicating that it has spatiotemporal characteristics. This atmospheric turbulence characteristic has similar effects on characteristic description parameters. However, the characteristics description parameters of wind speed and wind power variation fail to reflect such intrinsic characteristics that are not affected by the spatiotemporal diurnal modulation characteristics of atmospheric turbulence. This indicates that they do not have diurnal characteristics. Finally, a time-varying model combined with the spatiotemporal diurnal modulation characteristics of wind speed and its power generation is discussed by applying on the evaluation of frequency control in power systems. It is shown that the results obtained by measured data processing could improve the power generation quality of large-scale wind power effectively.

## 1. Introduction

As one of the renewable resources, wind energy is being used worldwide. Researchers from various countries have carried out lots of work on the current status and future of wind power in their countries [1–6]. Development of wind power from the viewpoints of political, social, and technical issues was analyzed in [7]. In China, wind power is the leading energy development sector under the low-carbon development policy, while a series of wind power development plans were drawn up by the government [8–10].

However, the uncertainty in wind speed poses challenges for the utilization of wind power [11–13]. In addition to randomness and volatility, intermittency is another problem that plagues the large-scale application of wind energy [14]. A series of strategies and paths covering the source sides, grid sides, and load sides have been studied and explored to use large-scale wind power safely and efficiently [2, 5, 15]. In [16], China's feed-in tariff mechanism for large-scale wind power is shown. Moreover, the characteristics of Chinese energy structure determine the developmental path of rapid and deep peak regulation of thermal power [17, 18]. Several novel methods were proposed for wind power to smooth the output power of wind energy [19, 20].

Considering the uncertainty of wind energy on grid connection, research work focuses on wind power prediction and how to do global dispatch planning and design by prediction results [6, 21, 22], including data model, physical model, and hybrid model [23–25]. The continuous development of artificial intelligence algorithms has further expanded the scope of its application to new areas [26, 27]. Using data obtained from data-rich farm, high-dimensional data features are obtained through a series of data extraction methods and applied to newly built farm. This transfer learning research is attracting further attention [28].

However, prediction error cannot be avoided due to wind speed uncertainty [29]. Further, quantitative characterization of the wind speed and generated power's persistence and variation is also urgently needed [30]. In view of the physical nature of the random fluctuation of wind speed and the characteristic of wind power, several studies have been carried out on their uncertainty, including instantaneous characteristics (power spectrum), short-period characteristics (daily variation), and long-period statistical characteristics.

In terms of the nature relationships of range to standard deviation of wind fluctuations, the fluctuation variance caused by turbulence is dependent on the mean wind speed per hour [31]. Literature [32] tries to predict the turbulence standard deviation of wind speed. Because of the importance of long-period characteristics, the spatiotemporal complementarity between solar and wind power in the Iberian Peninsula has been researched as the key problem [33, 34]. Additionally, wind speed variance is also an important parameter to characterize wind and turbulence intensity. Turbulence intensity can be used in fan safety design [35–37], life analysis [38–41], and wind farm layout design [42, 43] and, hence, has long been the focus of many researchers.

Although characterization of the wind resource is important [44], the most significant one is the intermittency caused by the inherent instability of atmospheric turbulence. In [45, 46], the measurement and analysis of intermittency for power generation and wind speed were performed based on historical data of wind speed and wind power. Although the intermittent characterization index of wind speed and power has the characteristics of daily cycle, neither the practical application of daily cycle nor the global perspective on the summarized daily modulation has been discussed.

In fact, due to the existence of the diurnal modulation of atmospheric turbulence, the diurnal periodic characteristics are reflected in the average wind speed and temperature [47, 48]. Many factors, such as atmospheric stability [49], time scale [48], near-surface temperature, and turbulence intensity [50], will all influence the wind characteristics. Therefore, modeling and research on diurnal cycle characteristics are also being carried out [51, 52].

In summary, the average wind speed followed by a certain law has been deeply studied presently. Scholars have studied several systematic algorithms on wind speed and its generated power prediction. However, only few studies study on turbulent wind speed due to the uncertainty caused by turbulence, and only a few researchers have conducted studies on turbulence intensity from the application aspects of wind turbines' life analysis and design of wind farm layout. Although several researches have pointed that the average wind speed, turbulence intensity, and temperature show diurnal characteristics, there is no study that has proposed the intrinsic characteristics of spatiotemporal diurnal modulation of atmospheric turbulence from a global perspective. Furthermore, there is also absence of a systematic analysis of the influences of diurnal modulation on wind speed and wind turbine randomness, volatility, and intermittency based on the essential characterization parameters of its output power.

In this paper, the spatiotemporal diurnal modulation characteristics of atmospheric turbulence and their influence on the indeterminacy of wind speed and power generation are discussed. The rest of this paper is organized as follows: in Section 2, the spatiotemporal diurnal modulation characteristics of atmospheric turbulence are proposed from a global perspective based on the analysis of its physical mechanism. In Section 3, the influence of spatiotemporal diurnal modulation of atmospheric turbulence on wind speed and its power production's random fluctuation range is analyzed based on their corresponding characterization index called the variance. In Section 4, the law of the influence of spatiotemporal diurnal modulation of atmospheric turbulence on the random fluctuation rate of speed is analyzed by variation index. Furthermore, this method is extended to analyze wind power fluctuation rate. In Section 5, the influence of wind speed and wind power intermittency on turbulent spatiotemporal daily modulation is analyzed based on the ramp duty ratio index. Moreover, another wind power intermittency characterization parameter without daily modulation is analyzed by the start/stop frequency of the wind turbines. In Section 6, considering the fluctuation range of wind speed and wind power as examples, the quantitative characterization modeling of the uncertainty and its introduction strategy in the evaluation of real-time frequency modulation capability of power grid are used to explore the feasibility of such spatiotemporal daily modulation characteristics. The seventh section is the conclusion and prospection of the paper.

## 2. Intrinsic Properties of Spatiotemporal Diurnal Modulation in Atmospheric Turbulence

### 2.1. Diurnal Modulation and Spatiotemporal Characteristics of Atmospheric Turbulence

Atmospheric turbulence is an intrinsic factor of uncertainty in wind speed and the power output of wind turbine. This uncertainty performance in atmospheric motion is attributed to the random fluctuation of various sizes superimposed by its average wind speed and wind direction. Similarly, it also applies to the power output of wind turbines.

Turbulence is a different kind of motion that takes place in the atmospheric boundary layer (ABL). The factors affecting its formation differ during day and night with apparent diurnal periodic characteristics. Generally, the turbine hub height is in the ABL. The diurnal variation of the sun rising in the east and setting in the west highly influences the convective motion of the ABL in a flat terrain where surface roughness is uniform. Therefore, diurnal variation can reflect the change in an underlying flat surface in the boundary layer. However, the turbulence intensity in the underlying surface layer of a complex mountainous area is affected by the vortex flow around the local landform, leading to the formation of intense turbulence as well, which is stronger than that caused by sunlight, where diurnal period characteristics are submerged. This influence, especially, is greater when the altitude closer to the ground, leading to a greater turbulence than that at a higher altitude. The diurnal periods of flat areas, such as plains or plateaus, have common features. For example, the time of maximum turbulence intensity is similar. Therefore, the diurnal period has spatiotemporal characteristics, which are derived from the intrinsic characteristics of the turbulence in ABL.

The process of controlling one parameter of a signal with another signal is defined as modulation in communication systems. Based on the influence of the diurnal period on turbulence and wind speed, this diurnal period can be considered as the modulation of wind speed. In other words, the diurnal period is a diurnal modulation process of wind speed caused by a longer weather process, and a diurnal modulation process of the atmosphere is the physical mechanism for the existence of diurnal period phenomenon in the turbulent part of wind speed and wind power. Therefore, several characterization indexes of wind speed uncertainty are affected by spatiotemporal diurnal modulation of atmospheric turbulence, such as the average wind speed of wind farm, the output power of the wind turbines, the random fluctuation part and internal intermittency, and the average temperature (heat flux) of the wind field. However, the descriptive parameters that cannot reflect the inherent characteristics of wind speed are not affected.

### 2.2. Other Recommendations

The scheduling, control, and planning arrangements in a power system have different time scales. The day-ahead prescheduling planning takes one day as a computation period, while the real-time scheduling and optimization control takes hours as the time scale. These correspond to the 0∼24 h day-ahead forecast and 0∼4 h ultra-short-term forecast of wind power, respectively. Therefore, if the parameters related to wind power prediction, such as the mean time value of wind speed and wind power, wind power uncertainty, and intermittency, especially for the hourly scale statistic rules, have diurnal modulation characteristics, it will have a guiding significance for the day-ahead prescheduling planning and real-time scheduling of primary and secondary frequency modulation in the new energy power system after large-scale wind power grid connection.

Autocorrelation analysis is a mathematical tool used to find repeated patterns and analyze value functions or sequences of signal processing, such as a periodic signal masked by noise. As for periodic sequences, the autocorrelation sequence shows periodic change [43].

## 3. Influence Law of Turbulence Diurnal Modulation on Wind Power Random Fluctuation Range

### 3.1. Quantitative Characterization of Wind Power Fluctuation Range

Generally, the actual wind speed can be divided into hourly average wind speed and turbulent wind speed based on Reynolds averaging. Research results show that the turbulent part of the wind speed depends on the average time strongly. A turbulence intensity model in international IEC standard realizes the wind speed fluctuation range (intensity). Subsequently, a universal model of turbulence intensity was proposed [43], as given by the following equation:

(1) $$\begin{array}{c} TI=\frac{\sigma }{\bar{\nu }}=\alpha \cdot {\bar{\nu }}^{-\beta }+c, \end{array}$$

where *σ* is variance of wind speed turbulence; $\bar{\nu }$ is the mean wind speed per hour; *α* , *β*, and *c* are constants. Similarly, the model of single power and relative variance for wind turbine or wind farm can be established. *I*_*p*_, the wind power fluctuation intensity, is the characteristic parameter of power fluctuation range [40]. The relative variance is the unit value of the residual fluctuation components after subtracting the mean value from the actual wind power. Based on the wavelet algorithm, the multiscale fluctuation intensity suitable for frequency modulation capability evaluation can be obtained as the minute-scale wind power fluctuation intensity *I*_*pm*_ and secondary wind power fluctuation intensity *I*_*ps*_, shown in equations (2) and (3), respectively.

(2) $$\begin{array}{c} I_{pm}=\frac{\sigma_{m}}{\bar{P}}=\alpha_{m}\times {\bar{P}}^{-\beta_{m}}+c_{m}, \end{array}$$

(3) $$\begin{array}{c} I_{ps}=\frac{\sigma_{s}}{\bar{P}}=\alpha_{s}\times {\bar{P}}^{-\beta_{s}}+c_{s}, \end{array}$$

where *σ*_*m*_ is the instantaneous standard deviation of wind power in minute-scale; *σ*_*s*_ is the instantaneous standard deviation of secondary wind power; $\bar{P}$ is the mean wind speed per hour; *α*_*m*_, *β*_*m*_ and *c*_*m*_ are the fitting constants of minute-scale wind power fluctuation intensity; *α*_*s*_, *β*_*s*_, and *c*_*s*_ are the fitting constants of secondary wind power fluctuation intensity.

### 3.2. The Influence Law on Wind Speed Fluctuation Range

There are many factors that affect the actual wind speed fluctuation uncertainty, and the three-parameter power law model also has a fitting error under certain conditions. The fitting error is defined as follows:

(4) $$\begin{array}{c} e=\sigma -\left(\alpha \cdot {\bar{\nu }}^{-\beta }+c\right)\bar{\nu }, \end{array}$$

where *σ* is the residual fluctuation standard deviation of the actual wind speed after eliminating average.

**Figure 1:**
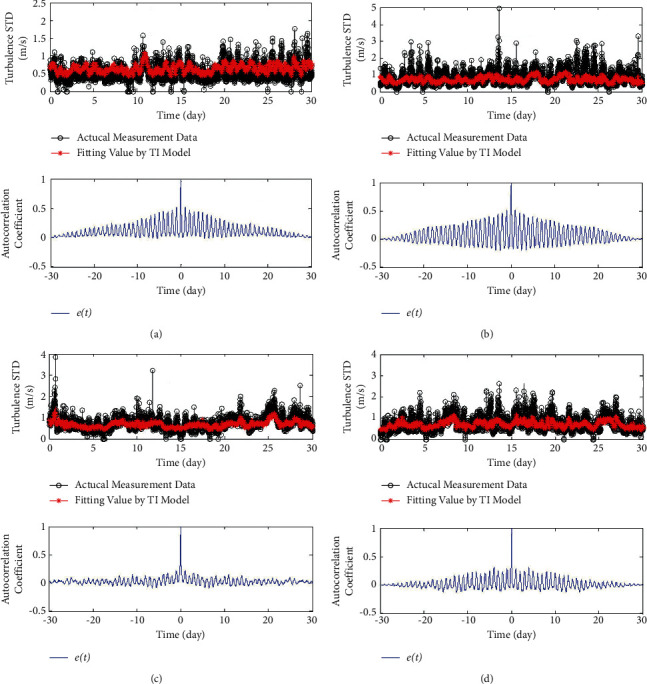
The fitting error of the model and its diurnal period pattern. (a) Wind data in spring. (b) Wind data in summer. (c) Wind data in autumn. (d) Wind data in winter.

Figure 1 shows the autocorrelation analysis of the fitting error. It can be seen that the fitting error has strong diurnal period characteristics, and the diurnal period change pattern is different for the four seasons.

**Figure 2:**
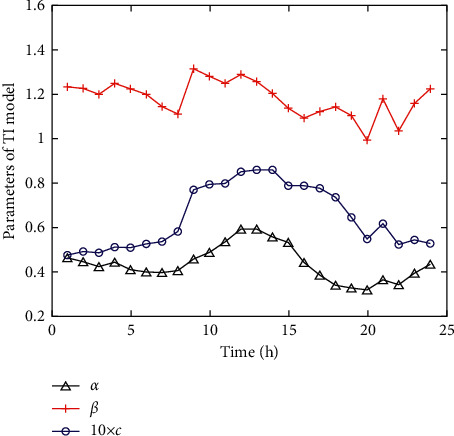
Model parameter variation within 24 h.

There is an obvious diurnal periodic change pattern of turbulence intensity combining the above analysis.  Figure 2 shows the variation law of the model parameters within 24 h obtained by fitting the data in Figure 1. It indicates that the model parameters are variable throughout the daily cycle, and the differences are relatively large, which cannot be ignored.

**Figure 3:**
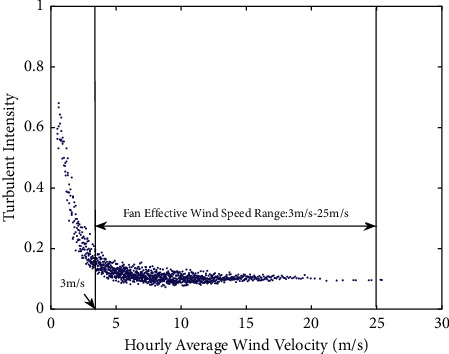
The hub height's average turbulence intensity.

Viewing from the perspective of wind turbine scheduling and controlling, the average turbulence intensity is defined by the effective section in the middle of the cut-in/cut-out speed of the universal fan. As shown in Figure 3, the average turbulence intensity in the wind speed section, ranging from 3 m/s to 25 m/s, corresponds to the hub height of the fan. The calculation used is shown in the following equation:

(5) $$\begin{array}{c} \bar{I}=\frac{1}{n}\sum_{i=1}^{n}I_{i}, \end{array}$$

where *I*_*i*_ is the turbulence intensity corresponding to the average speed range from 3 m/s to 25 m/s; *n* is the number of samples within the average speed range from 3 m/s to 25 m/s.

**Figure 4:**
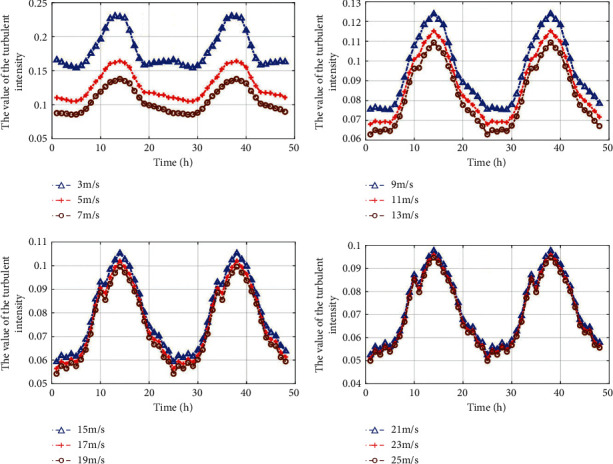
The variation of turbulence intensities for different wind speeds within 24 h using fitting model.

**Figure 5:**
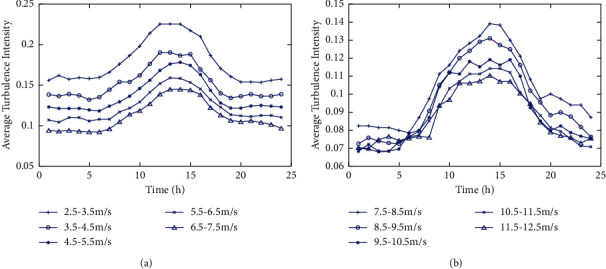
The variation of the mean turbulence intensities value within 24 h time interval (actual data). (a) Wind speed range from 2.5 m/s to 7.5 m/s. (b) Wind speed range from 7.5 m/s to 12.5 m/s.

Figure 4 shows the diurnal period variation pattern of different wind speed turbulence intensities obtained by the fitting model.  Figure 5 shows the diurnal period patterns of different wind speed turbulence intensities obtained from the measured data. All of them prove the existence of the diurnal period change pattern of turbulence intensity.

**Figure 6:**
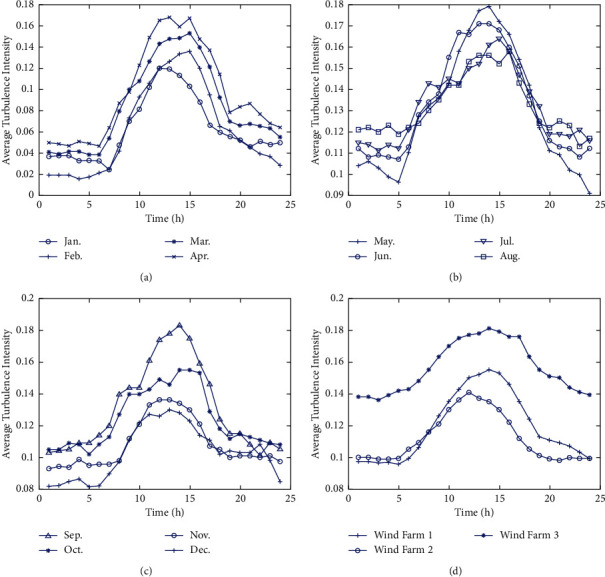
The diurnal cycle variation of TI during different seasons and months of a year. (a) The diurnal cycle from January to April. (b) The diurnal cycle during May and August. (c) The diurnal cycle from September to December. (d) The diurnal cycle variation of mean TI in three different farms.

Figures 6(a)–6(c) show seasonal and monthly variation patterns of turbulence intensity in diurnal period, including diurnal variation patterns of turbulence intensity in four seasons and twelve months. It can be seen that the diurnal period pattern of turbulence intensity in this region is affected by the season and month. Generally, the average turbulence intensity is the highest in spring and the lowest in winter. The maximum value of average turbulence intensity in a single month occurs in May and September. In terms of the 24 h variation in a day, the maximum value occurs around 14 : 00∼15 : 00, and the minimum value occurs around 20 : 00 of the same day∼08 : 00 of the next day. The maximum value occurs earlier in winter compared to other seasons.

All the investigated wind farms were located in typical areas of northern China, but the diurnal cycle patterns of the actual offshore wind farms were different. Land turbulence intensity whose driving energy is predominantly from the sun has a diurnal cycle variation pattern, and the difference is mainly due to the difference in light intensity and illumination time during different seasons and months. It means that the diurnal modulation of atmospheric turbulence has spatiotemporal characteristics.

To further illustrate the issue, the one-year data from three wind farms in different regions are analyzed based on effective average turbulence intensity parameters.  Figure 6(d) shows the diurnal characteristics of average turbulence intensity in different regions. The diurnal cycle patterns of three wind farms differ with the time and location. The main reason for this is the wind farms located in the different latitudes and longitudes. It means that the time of sunrise and sunset is different, resulting in a time deviation of the maximum value.

**Figure 7:**
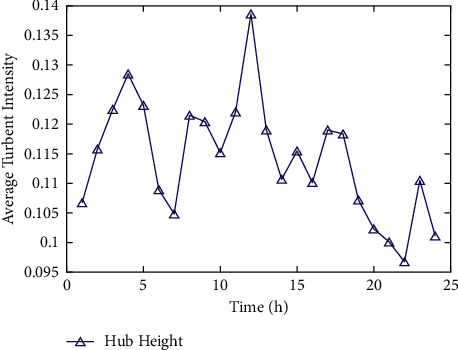
The diurnal cycle variation of TI in land farm with complex terrain.

**Figure 8:**
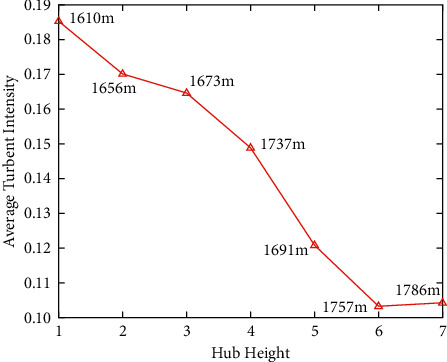
The diurnal cycle variation of TI in land farm with complex terrain.

As shown in Figure 7, the influence of the sunshine on the diurnal variation is weakened by complex topography and varied surface roughness.  Figure 8 shows the maximum value of the average turbulence intensity corresponding to the fan at the lowest altitude is nearly twice that of its maximum value corresponding to the fan at the highest altitude. Therefore, complex topography has a great influence on the average turbulence intensity. The lower the altitude, the greater it is affected by topography and roughness. However, literature [43] does not elaborate on these aspects.

### 3.3. The Influence Law of Turbulence Diurnal Modulation on Wind Power Fluctuation Range

**Figure 9:**
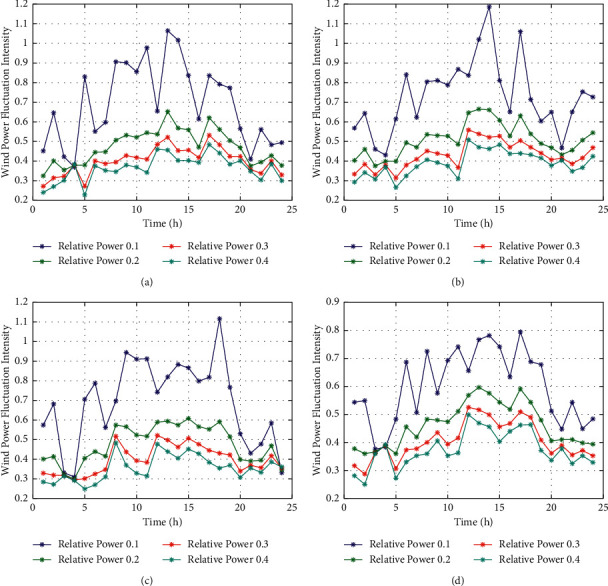
The diurnal cycle of wind power variance in same wind field. (a) #19 turbine. (b) #39 turbine. (c) #69 turbine. (d) #119 turbine.

The diurnal cycle characteristics of wind power fluctuation intensity are not mentioned in the existing literature.  Figure 9 shows the diurnal cycle characteristics of wind power fluctuation intensity of the different fans in the same wind field. Due to the variance of wind speed has diurnal cycle characteristics, the variance of wind power also has diurnal cycle characteristics. Moreover, the wind power fluctuation intensity corresponding to different power values also has diurnal cycle characteristics. However, after the conversion of wind speed into energy by means of the fan, the regularity of the diurnal cycle change of wind power fluctuation intensity is not as apparent as that of the diurnal cycle change of wind speed turbulence intensity.

## 4. Influence Law of Turbulence Diurnal Modulation on Wind Power Random Fluctuation Rate

### 4.1. Quantitative Characterization of Wind Power Fluctuation Rate

Variational analysis is introduced in [41] to establish a computing model of instantaneous wind speed variation based on wavelet algorithm. In addition, the quantitative characterization of wind speed fluctuation rate is realized based on the dependence of wind speed variation on hourly average wind speed, similar to turbulence intensity. The variation of wind power also has the same characteristics. We further defined the variable intensity of wind power *χ*_*p*_ as the derived parameter to describe wind power variation quantitatively, as given by the following equation:

(6) $$\begin{array}{c} \chi_{p}=\frac{{\left[\gamma_{p}^{*}\left(\Delta t\right)\right]}^{1/2}}{{\bar{p}}^{*}}=\alpha \cdot {\left({\bar{p}}^{*}\right)}^{-\beta }+c, \end{array}$$

where *γ*_*p*_^*∗*^(Δ*t*) is the relative variation of wind power; ${\bar{p}}^{*}$ is the unit value of the average hourly power; *α*, *β*, and *c* are fitting constants.

### 4.2. Influence Law of Turbulence Diurnal Modulation on Wind Speed Fluctuation Rate

**Figure 10:**
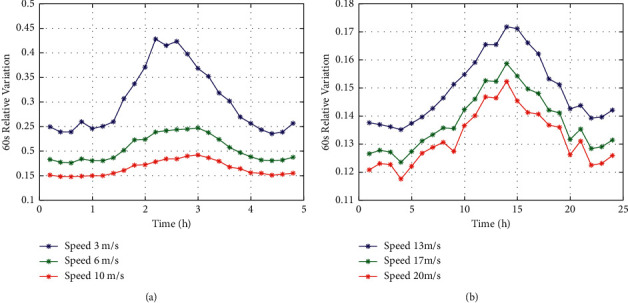
The diurnal cycle of different wind speed variation. (a) Wind speed: 3 m/s, 6 m/s, 10 m/s. (b) Wind speed: 13 m/s, 17 m/s, 20 m/s.

In [41], one-month time series data was used for autocorrelation analysis, which adopted multitime interval variation calculation. The results show that the time lag of the variation function is a periodic component in the time series, and the variation period is 1 day. The regularity of diurnal cycle change pattern remains constant with an increase in time interval. The diurnal cycle regularity becomes weaker only when the time interval reaches a certain level. The variation function of wind speed has the same diurnal cycle pattern as the variance. The time series *γ*(Δ*t*), as in [41], has different time lags, and the diurnal pattern of wind speed variation function is analyzed in detail. *γ*(Δ*t*) is large from 8 a.m. to 6 p.m., especially at its peak between 12 a.m and 4 p.m, and relatively small during other times of the day. On the basis of [41], further analysis reveals that diurnal cycle characteristics are not related to the speed, and speeds at different sizes have similar daily cycle characteristics shown in Figure 10.

### 4.3. The Diurnal Period Characteristics of Wind Power Variogram

**Figure 11:**
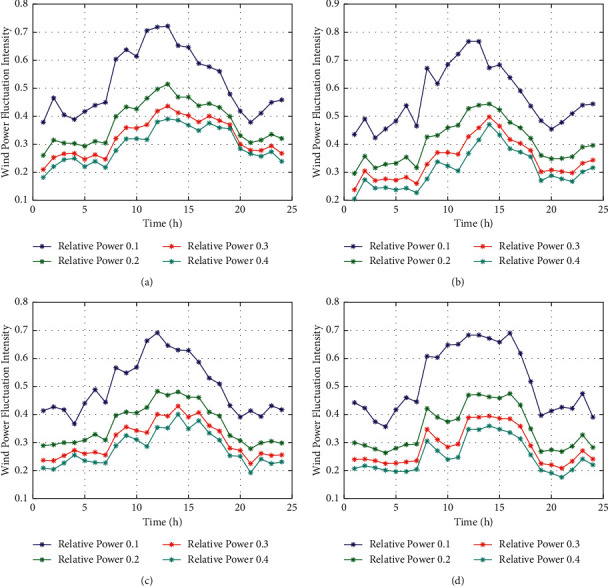
The diurnal cycle of different wind turbine power variogram in wind field. (a) #19 turbine. (b) #39 turbine. (c) #69 turbine. (d) #119 turbine.

Based on [41], the universality of the diurnal period characteristics of wind power variogram is further analyzed. As shown in Figure 11, the measured wind power data of multiple Wind generators are selected. It shows that wind power variogram is modulated by the diurnal process and has an obvious diurnal period characteristics. However, none of the present literature mentions the diurnal cycle characteristics of wind power variogram. Therefore, we extend the results of our research from characteristics of wind speed to its producing wind power. In power grid dispatching, the wind speed during noon has strong fluctuations at a fast rate. Therefore, when the disturbance caused by the power stroke of the grid is suppressed, it is necessary to reasonably configure fans with different suppression capacities to ensure safe and stable operation of the grid.

## 5. Influence Law of Turbulence Diurnal Modulation on Wind Power Intermittency

In [45], a quantitative description method of intermittency is proposed based on the duty ratio of abrupt change in wind speed, which considers the atmospheric turbulence physical essence. Furthermore, in [46], this kind of quantitative description method is extended to the definition of wind power intermittency, which is of great significance to the power system.

In [45, 46], similar autocorrelation has been adopted to analyze the measurement parameters of wind speed and its generating power intermittently in detail. It was found that the abrupt duty cycle parameters of wind power had obvious diurnal cycle characteristics. Similar to the diurnal cycle of wind power variance and variogram parameters, the steep duty ratio between 8 a.m. and 8 p.m. is larger than other times. It reaches its daily peak between 12 a.m and 4 p.m. With similar statistical analyze methods, the results calculated on sufficient data from this farm but other months and other wind farms are identical. A study on the daily-cycle characteristic of the abrupt duty cycle of wind speed at different time intervals showed that the diurnal cycle phenomenon of the abrupt duty cycle of wind speed still exists. This proves the universality of the diurnal cycle phenomenon. The abrupt change in the diurnal cycle phenomenon of wind speed in duty cycle indicates that the wind power intermittency is stronger during the day than during night. Wind power intermittency reaches its daily peak between 12 a.m and 4 p.m. Therefore, the intermittent characterization index reflects the intrinsic characteristics of wind power uncertainty.

Although several parameters are used to characterize wind power intermittency, these parameters that cannot describe the nature of the intermittent wind speed and atmospheric turbulence also have no modulation effect.

## 6. Applications

### 6.1. Improved Wind Power Uncertainty Model Considering the Diurnal Modulation Characteristics of Atmospheric Turbulence

The study of the diurnal modulation characteristics of atmospheric turbulence on wind power uncertainty has the following practical significances for the utilization of wind power:

1. The diurnal period characteristics indicate that some factors of turbulence will affect the parameters of wind power variance and variogram model. Therefore, the region, topography, seasons, and weather patterns (such as sunny and rainy weather) will influence atmospheric turbulence.
2. The diurnal period of wind speed variance is a reflection of the random fluctuation of wind speed. Therefore, wind speed fluctuation range should be larger when turbulence intensity is higher, instead of a fluctuation range of equal widths.
3. According to the diurnal period of the wind speed variogram, the wind power change rate is unstable at different times during a day, which means that system operators must change their strategies in different states. More specifically, complementary power and control strategies that respond faster must be provided to ensure the safe and efficient operation of the new energy power system when the light intensity is stronger.
4. In addition, the diurnal cycle characteristics of wind speed variance and variogram also have reference value of the electricity price bidding for wind farm. The diurnal cycle characteristics of intermittency indexes have a similar significance for the operation of wind farms [53].

The diurnal cycle characteristics of wind power have a great impact on the precision of qualitative model fitting. According to the fitting effect of time-sharing model in [43], we can further optimize the variation and intermittency models. Taking the variance model as an example, the model considering time-varying parameters has the higher fitting accuracy [54]. The time-varying parameter model is shown in the following equation:

(7) $$\begin{array}{c} \frac{\sigma \left(t\right)}{\bar{\nu }\left(t\right)}=\alpha \left(t\right)\cdot \bar{\nu }{\left(t\right)}^{-\beta \left(t\right)}+c\left(t\right), \end{array}$$

where *σ*(*t*) is the variance of actual measurement wind speed; $\bar{\nu }\left(t\right)$ is the mean actual wind speed per hour; *α*(*t*), *β*(*t*), and *c*(*t*) are a group of constants obtained by data fitting and time-sharing modeling.

**Figure 12:**
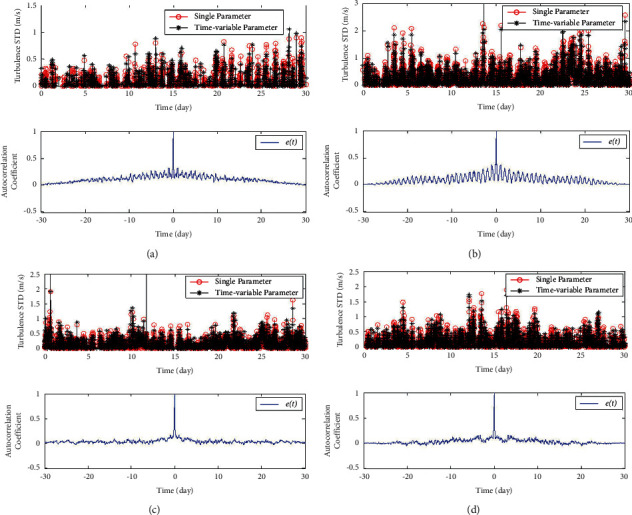
The fitting error of time variant model considering daily cycle model. (a) Wind data in spring. (b) Wind data in summer. (c) Wind data in autumn. (d) Wind data in winter.

As shown in Figure 12, the fitting error of this model considers the characteristics of diurnal cycle. Compared with Figure 1, the results show that this model has a great fitting accuracy rather than the original one (equation (1)).

The following four indicators of quantitative fitting effect are statistically analyzed.

(8) $$\begin{array}{c} \text{MNRE}=\frac{1}{n}\overset{n}{\underset{i=1}{\sum }}\left|\frac{\left(y_{ri}-y_{fi}\right)}{y_{ri}}\right|,\\ \text{MNSE}=\frac{1}{n}\overset{n}{\underset{i=1}{\sum }}\left|\frac{{\left(y_{ri}-y_{fi}\right)}^{2}}{y_{ri}^{2}}\right|,\\ \text{HASL}=k_{\left(\rho_{k}>0.6\right)},\\ \text{MAC}=\frac{1}{n}\overset{n}{\underset{k=1}{\sum }}\left|\rho_{k}-\min\left(\rho_{k}\right)\right|, \end{array}$$

where MNRE and MNSE are mean normalized relative error and mean normalized square error, respectively. *y*_*ri*_ and *y*_*fi*_ are the actual variance values calculated by using wind speed data and obtained by using model fitting, respectively. HASL and MAC are the mean of the highly autocorrelated step size and autocorrelation coefficient, respectively. *ρ*_*k*_ is the autocorrelation coefficient. *k*_(*ρ*_*k*_ > 0.6)_ is the step size corresponding to *ρ*_*k*_ > 0.6.

Table 1 shows a comparison of the results. It can be seen that the fitting-effect of the time-varying parameter model is better than that of the original model.

### 6.2. Example Analysis of Frequency Modulation Capability Estimation Considering the Diurnal Characteristic of Wind Power Fluctuation Intensity

**Figure 13:**
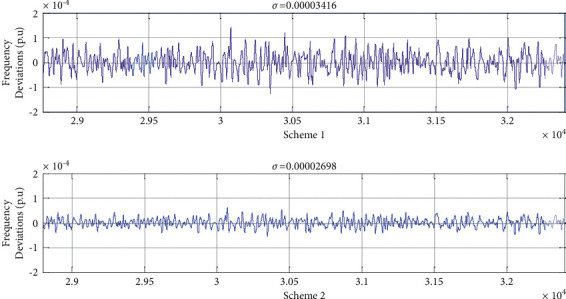
Comparison of simulation results of two different schemes.

According to [40], the fluctuation range measurement of wind power can be analyzed combined with the primary frequency modulation (PFM) and secondary frequency modulation (SFM) characteristics of thermal power units. The units for frequency modulation can be rationally configured to meet the analysis requirements of frequency modulation. The fluctuation intensity of minute and second wind power in a wind farm during a day was fitted, and the corresponding fluctuation time series were calculated as follows:

1. When curve fitting is carried out at all time periods, the corresponding single-fitting parameter in equation (2) can be obtained. Based on the fitting model, wind power fluctuations in different time scales are estimated. The allocation scheme 1 is as follows: the proportion of primary FM unit is 80%. The inequality of turbine generator unit *δiA* = 0.05. The proportion of secondary FM unit is 40%, and the integral gain KA of secondary FM channel is 0.25. At this time, formula 20 in reference [40] (DFPRA) of the primary frequency modulation capability of the system increases from 7.9523 to 15.9045, and formula 21 in reference [40] (DSPR) of the secondary frequency modulation capability increases from 11.9636 to 23.9273.
2. According to the diurnal modulation characteristics, the data are fitted during a day. The corresponding power law model of wind power fluctuation in this period is obtained subsequently. Based on the law model of time-sharing wind power fluctuation, the wind power fluctuation during this period can be estimated accurately. So, scheme 2 is as follows: the primary FM unit of share A is increased from 40% to 50%. The range of turbo-generator set rate *δiA* is reduced from 0.05 to 0.025. The secondary FM unit share is increased from 20% to 40%. The integrator gain KA is increased from 0.25 to 0.5. Such a system has the primary frequency capacity DFPRA increasing from 7.9523 to 19.9004, and secondary frequency modulation capacity DSPRA increased from 11.9636 to 60.0533. The system's frequency deviation after adopting the two schemes is shown in Figure 13. It can be seen that the standard deviation of the frequency fluctuation of the system *σ* = 0.00003416 when scheme 1 is used in region A being reduced to *σ* = 0.00002698 when scheme 2 is used. Therefore, the time-sharing modeling method, considering the daily modulation characteristics, can achieve accurate estimation of wind power fluctuations, realize the optimal allocation of FM units, and provide a reference for the power grid dispatchers to rationally allocate FM units.

## 7. Conclusion

Considering the physical nature of atmospheric motion, the wind power uncertainty is studied in the present research. The influence of the diurnal cycle characteristic of atmospheric turbulence on the fluctuation of wind speed and wind power is studied in this paper. The results show that this characteristic itself is affected by season, latitude, longitude, and topography. However, its influence on wind power fluctuation is weakened after the energy conversion process. Then, we analyze the influence of the diurnal cycle characteristic on the random fluctuation rate of wind power, and wind power intermittency. The results show that this parameter has no influence on the wind speed and the proportion of wind speed steepness but affects the duty ratio of abrupt change in wind speed.

Based on this research, we propose a time-varying model considering the diurnal cycle characteristic, and the fitting error of wind speed and its power generation fluctuation models are all smaller compared with the original models. At the same time, the wind power fluctuation is estimated based on the law model of time-sharing wind power fluctuation. In the two simulation schemes, the standard deviation of the system frequency fluctuation is reduced to 0.00003416 and 0.00002698, respectively, which verifies the validity of the model.

This paper presented the spatiotemporal diurnal modulation characteristics of atmospheric turbulence from the perspective of real-time scheduling and optimal control of power systems. This is of practical significance for power grid dispatchers to adjust the frequency modulation resources in real time and ensure the safety and reliability of system frequency.

## Figures and Tables

**Table 1:** Statistical analysis of the fitting error.

Dataset	Original model				Time-varied model			
	MNRE(%)	MNSE(%)	HASL	MAC	MNRE(%)	MNSE(%)	HASL	MAC
1	47.53	13.90	8	0.1222	38.78	9.56	3	0.1091
2	36.12	15.21	8	0.2661	28.40	10.44	2	0.0943
3	27.73	7.68	2	0.1181	25.11	6.73	1	0.0658
4	33.41	10.87	5	0.1493	27.71	7.88	2	0.0853

## Data Availability

The data used to support the findings of this study are available from the corresponding author upon request.

## References

[1] Scarabaggio P., Grammatico S., Carli R., Dotoli M. (2022). Distributed demand side management with stochastic wind power forecasting. *IEEE Transactions on Control Systems Technology*.

[2] Zhang H., Hu X., Cheng H. (2021). Coordinated scheduling of generators and tie lines in multi-area power systems under wind energy uncertainty. *Energy*.

[3] Wu C., Luo K., Wang Q., Fan J. (2022). A refined wind farm parameterization for the weather research and forecasting model. *Applied Energy*.

[4] Tsao H. H., Leu Y. G., Chou L. F. (2021). A center-of-concentrated-based prediction interval for wind power forecasting. *Energy*.

[5] Lyu X., Jia Y., Liu T., Chai S. (2021). System-oriented power regulation scheme for wind farms: the quest for uncertainty management. *IEEE Transactions on Power Systems*.

[6] Lu P., Ye L., Zhao Y., Dai B., Pei M., Tang Y. (2021). Review of meta-heuristic algorithms for wind power prediction: methodologies, applications and challenges. *Applied Energy*.

[7] Juárez A. A., Araújo A. M., Rohatgi J. S. (2014). Development of the wind power in Brazil: Political, social and technical issues. *Renewable and Sustainable Energy Reviews*.

[8] Zhao Z. Y., Wu P. H., Xia B. (2014). Development route of the wind power industry in China. *Renewable and Sustainable Energy Reviews*.

[9] Shen J., Song X., Ming Z., Yi W., Yuejin W., Xiaoli L. (2014). Low-carbon development strategies for the top five power generation groups during China׳s 12th Five-Year Plan period. *Renewable and Sustainable Energy Reviews*.

[10] Baichen X., Lifeng S., Wenhua L., Lu Z.-Y. (2015). Study on China’s wind power development path Based on the target for 2030. *Renewable and Sustainable Energy Reviews*.

[11] Slootweg J. G., Kling W. L. (2003). The impact of large scale wind power generation on power system oscillations. *Electric Power Systems Research*.

[12] Makarov Y. V., Loutan C., Ma J. (2009). Operational impacts of wind generation on California power systems. *IEEE Transactions on Power Systems*.

[13] Callegari G., Capurso P., Lanzi F., Merlo M., Zaottini R. Wind power generation impact on the frequency regulation: study on a national scale power system.

[14] Ren G., Liu J., Wan J., Guo Y. (2017). Overview of wind power intermittency: impacts, measurements, and mitigation solutions. *Applied Energy*.

[15] Wu J., Wang Z. X., Wang G. Q. (2014). The key technologies and development of offshore wind farm in China” Renewable and Sustainable. *Energy Reviews*.

[16] He Y., Pang Y., Zhang J., Xia T., Zhang T. (2015). Feed-in tariff mechanisms for large-scale wind power in China. *Renewable and Sustainable Energy Reviews*.

[17] Wang W., Zeng D., Liu J., Niu Y., Cui C. (2014). Feasibility analysis of changing turbine load in power plants using continuous condenser pressure adjustment. *Energy*.

[18] Wang W., Jing S., Sun Y., Niu Y., Zeng D., Cui C. (2019). Combined heat and power control considering thermal inertia of district heating network for flexible electric power regulation. *Energy*.

[19] Howlader A. M., Urasaki N., Yona A., Senjyu T., Saber A. Y. (2013). A review of output power smoothing methods for wind energy conversion systems. *Renewable and Sustainable Energy Reviews*.

[20] Mahela O. P., Shaik A. G., Kazmerski L. (2016). Comprehensive overview of grid interfaced wind energy generation systems. *Renewable and Sustainable Energy Reviews*.

[21] Wang J., Song Y., Feng L., Hou R. (2016). Analysis and application of forecasting models in wind power integration: a review of multi-step-ahead wind speed forecasting models. *Renewable and Sustainable Energy Reviews*.

[22] Ssekulima E. B., Anwar M. B., Hinai A. A., Moursi M. S. E. (2016). Wind speed and solar irradiance forecasting techniques for enhanced renewable energy integration with the grid: a review. *IET Renewable Power Generation*.

[23] Al-Yahyai S., Charabi Y., Gastli A. (2010). Review of the use of numerical weather prediction (NWP) models for wind energy assessment. *Renewable and Sustainable Energy Reviews*.

[24] Yao Z., Wang J., Wang X. (2014). Review on probabilistic forecasting of wind power generation. *Renewable and Sustainable Energy Reviews*.

[25] Shi J., Guo J., Zheng S. (2012). Evaluation of hybrid forecasting approaches for wind speed and power generation time series. *Renewable and Sustainable Energy Reviews*.

[26] Lecun Y., Bengio Y., Hinton G. (2015). Deep learning. *Nature*.

[27] Wan J., Liu J., Ren G., Hu Q. (2016). Day-ahead prediction of wind speed with deep feature learning. *International Journal of Pattern Recognition and Artificial Intelligence*.

[28] Hu Q., Zhang R., Zhou Y. (2016). Transfer learning for short-term wind speed prediction with deep neural networks. *Renewable Energy*.

[29] Bludszuweit H., Domínguez-Navarro J. A., Llombart A. (2008). Statistical analysis of wind power forecast error. *IEEE Transactions on Power Systems*.

[30] Li J. H., Li J. M., Wen J. Y., Yue C. (2014). Generating wind power time series based on its persistence and variation characteristics. *Science China Technological Sciences*.

[31] Markee E. H. (1963). On the relationships of range to standard deviation of wind fluctuations. *Monthly Weather Review*.

[32] Ren G., Liu J., Wan J. (2018). Prediction of the wind speed turbulence standard deviation. *Journal of Environmental Informatics*.

[33] Hill D. C., Mcmillan D., Bell K. R. W., Infield D. (2011). Application of auto-regressive models to U.K. Wind speed data for power system impact studies. *IEEE Transactions on Sustainable Energy*.

[34] Jerez S., Trigo R. M., Sarsa A., Montavez J. P. (2013). Spatio-temporal complementarity between solar and wind power in the iberian Peninsula. *Energy Procedia*.

[35] Barthelmie R. J., Frandsen S. T., Nielsen M. N., Pryor S. C. (2010). Modelling and measurements of power losses and turbulence intensity in wind turbine wakes at Middel grunden offshore wind farm. *Wind Energy*.

[36] Früh W. G., Creech A. C. W., Maguire A. E. (2014). Turbulence characteristics in offshore wind farms from LES simulations of lillgrund wind farm. *Energy Procedia*.

[37] Gualtieri G. (2015). Surface turbulence intensity as a predictor of extrapolated wind resource to the turbine hub height. *Renewable Energy*.

[38] Geng X., Zhou L., Freedman J. M., Cervarich M. C. (2016). A case study of effects of atmospheric boundary layer turbulence, wind speed, and stability on wind farm induced temperature changes using observations from a field campaign. *Climate Dynamics*.

[39] Bardal L. M., STran L. R. (2017). Influence of turbulence intensity on wind turbine power curves. *Energy Procedia*.

[40] Guo Y., Wang Q., Zhang D., Yu J. (2018). A stochastic-process-based method for assessing frequency regulation ability of power systems with wind power fluctuations. *Journal of Environmental Informatics*.

[41] Liu J., Ren G., Wan J., Guo Y., Yu D. (2016). Variogram time-series analysis of wind speed. *Renewable Energy*.

[42] Cheynet E., Jakobsen J. B., Obhrai C. (2017). Spectral characteristics of surface-layer turbulence in the North Sea. *Energy Procedia*.

[43] Ren G., Liu J., Wan J., Li F., Yu D. (2018). The analysis of turbulence intensity based on wind speed data in onshore wind farms. *Renewable Energy*.

[44] Cosseron A., Gunturu U. B., Schlosser C. A. (2013). Characterization of the wind power resource in europe and its intermittency. *Energy Procedia*.

[45] Ren G., Liu J., Wan J., Guo Y., Liu J. (2016). Measurement and statistical analysis of wind speed intermittency. *Energy*.

[46] Ren G., Wan J., Liu J., Yu D. (2018). Analysis of wind power intermittency based on historical wind power data. *Energy*.

[47] Qu M., Wan J., Hao X. (2014). Analysis of diurnal air temperature range change in the continental United States. *Weather & Climate Extremes*.

[48] Chien H., Cheng H. Y., Yang K. H., Chang W. T. (2015). Diurnal and semidiurnal variability of coastal wind over Taiwanese waters. *Wind Energy*.

[49] Wharton S., Lundquist J. K. (2010). *Assessing Atmospheric Stability and the Impacts on Wind Characteristics at an Onshore Wind Farm*.

[50] Smith C. M., Barthelmie R. J., Pryor S. C. (2013). In situ observations of the influence of a large onshore wind farm on near-surface temperature, turbulence intensity and wind speed profiles. *Environmental Research Letters*.

[51] Akinnubi R. T., Adeniyi M. O. (2018). Modeling of diurnal pattern of air temperature in a tropical environment: ile-Ife and Ibadan, Nigeria. *Modeling Earth Systems & Environment*.

[52] Kanamori H., Yasunari T., Kuraji K. (2013). Modulation of the diurnal cycle of rainfall associated with the MJO observed by a dense hourly rain gauge network at sarawak, borneo. *Journal of Climate*.

[53] Li C., Peng H., Sun J. (2016). Predictive control and sizing of energy storage to mitigate wind power intermittency. *Wind Energy*.

[54] Wan J., Fan S., Yao K., Han K., Peng E., Yu J. (2021). Spatiotemporal diurnal modulation characteristic of wind speed and power generation revealed by its actual measurement data information processing. *Research Square*.

